# How to set up and manage a trainee-led research collaborative

**DOI:** 10.1186/1472-6920-14-94

**Published:** 2014-05-14

**Authors:** George Dowswell, David C Bartlett, Kaori Futaba, Lisa Whisker, Thomas D Pinkney

**Affiliations:** 1 Primary Care Clinical Sciences, University of Birmingham, Edgbaston, Birmingham B15 2TT, UK; 2 West Midlands Research Collaborative (WMRC), Birmingham, UK

## Abstract

**Background:**

Ensuring that doctors in training acquire sufficient knowledge, experience and understanding of medical research is a universal and longstanding issue which has been brought into sharper focus by the growth of evidence based medicine. All healthcare systems preparing doctors in training for practice have to balance the acquisition of specific clinical attitudes, knowledge and skills with the wider need to ensure doctors are equipped to remain professionally competent as medical science advances. Most professional medical bodies acknowledge that this requires trainee doctors to experience some form of research education, not only in order to carry out original research, but to acquire sufficient academic skills to become accomplished research consumers in order to remain informed throughout their professional practice. There are many barriers to accomplishing this ambitious aim.

**Discussion:**

This article briefly explains why research collaboratives are necessary, describes how to establish a collaborative, and recommends how to run one. It is based on the experiences of the pioneering West Midlands Research Collaborative and draws on the wider literature about the organisation and delivery of high quality research projects. Practical examples of collaborative projects are given to illustrate the potential of this form of research organisation.

**Summary:**

The new trainee-led research collaboratives provide a supportive framework for planning, ownership and delivery of high quality multicentre research. This ensures clinical relevance, increases the chances of research findings being translated into changes in practice and should lead to improved patient outcomes. Research collaboratives also enhance the research skills and extend the scientific horizons of doctors in training.

## Background

Ensuring that doctors in training acquire sufficient knowledge, experience and understanding of medical research is a universal and longstanding issue [1] which has been brought into sharper focus by the growth of evidence based medicine [2]. All healthcare systems preparing doctors in training for practice have to balance the acquisition of specific clinical attitudes, knowledge and skills with the wider need to ensure doctors are equipped to remain professionally competent as medical science advances. Most professional medical bodies acknowledge that this requires trainee doctors to experience some form of research education, not only in order to carry out original research, but to acquire sufficient academic skills to become accomplished research consumers in order to remain informed throughout their professional practice. There are many barriers to accomplishing this ambitious aim. Doctors in training are occupied with clinical practice, especially in a ‘craft’ speciality such as surgery where a practical skill-set must be acquired in addition to the knowledge-base. Furthermore, the European Working Time Directive has reduced working hours making it a challenge to meet clinical training requirements, let alone research. Trainees may lack interest, resources, access to appropriate teaching or inspirational role models. Universities may fail to offer educational activities which are timely, accessible and adaptable. In the UK at least, it is still the case that a higher degree is a necessity in order to progress within the more highly competitive specialties, including many surgical specialties. Therefore some trainees experience a period of research as a means to an end without really embracing the process and benefiting from it. For those who do wish to undertake a period of formal research training, the new clinical training pathways limit options and make it increasingly difficult to obtain time for research. Funding for research training posts is extremely competitive and obtaining funding as an individual outside of such a programme is perceived as almost impossible. A US survey of residency program directors in 2006 reported that almost all (95%) required a research project to be completed but that less than half provided teaching on clinical trial design, biostatistics or access to essential statistical software [3]. With inadequate support, many doctors in training are put off the idea of research altogether, or commence research projects which are impractical, over-ambitious or inappropriate, frequently leading to failure and an aversion towards further research. Resulting research outputs are either not disseminated or unlikely to contribute to science or influence practice (too small, single centre, lacking in follow up, not generalisable) [4].

There is evidence that positive research experiences of trainee doctors can have a lasting beneficial effect on their subsequent careers [5]. This might be reflected in lifelong academic inquisitiveness, active research interests or translated into considerable contributions to teaching. All these outcomes could improve patient care.

Traditional educational models based on individual examination and assessment are potentially in conflict with the growing recognition of science as a social undertaking [6]. Underpowered single centre studies with small samples and large confidence intervals are becoming increasingly unacceptable to funders [7]. Scientific work (including biomedicine) is increasingly being done by collaborative networks [8], and the potential for (so called) ‘collaboratories’ has been extolled [9], but not always realised [10]. Collaboratories use information and communication technology to enable geographically dispersed researchers to address common interests by giving access to the data, artefacts or tools needed to accomplish research [11]. Collaboration requires additional communication, shared working practices and common goals which may be absent from daily practice [10]. Working across institutions has created problems for scientists – issues of ownership, funding, control and intellectual property are challenges for many projects [12]. There are also well documented difficulties associated with working across geographical distance – trust [13], misunderstandings [14], communication [15], and assumptions [16] have all been identified as major challenges.

### The research collaborative model

For the reasons discussed, a forum in which trainees are able to engage in research with the advice and support of experienced researchers and the flexibility to allow research to be combined with training commitments could be beneficial. Such a venture should allow all trainees within a region or training group to be involved, whether they have a longer term academic interest or not, and allow them to contribute towards projects as and when they have time throughout the entire period of training regardless of individual placements. Skills and attitudes which will benefit future careers should be developed, as well as tangible benefits such as publications. The research collaborative aims to fulfil these needs (and increase access to research funding opportunities). In this article we will discuss how to set up and run such a collaborative, and describe both our experiences and some of our achievements.

## Discussion

### How to establish a research collaborative

There is some evidence in the literature of how to start (and successfully complete) a collaborative project [17], but this tends to come from personal experience rather than systematic studies. More scholarly treatment has been given to identifying the barriers to collaboration [18]. Comparative retrospective analysis of data from a large scale evaluation of physics collaborations considered seven dimensions and their impact on stress, conflict and perceived success – project formation and compositions, magnitude, interdependence, communication, bureaucracy, participation and technological practice. However, no strong relationships were found for most of these factors [19]. It has been speculated that more basic factors – high levels of interest, adequate funding and tangible outputs may have been most important features of successful projects [10]. In effect, the literature on non-medical scientific collaborations suggests that interest is not a problem; initial funding is less important than leadership and commitment by senior clinicians; outputs take time to emerge but success breeds success [17-20].

The type of trainee-led research collaboratives described in this article are essentially complex groups (from multiple administrative units – hospitals in a geographical region) with a degree of academic heterogeneity (trialists, health economists, and clinicians, working jointly from discipline specific bases – inter-disciplinarity) [21].

As a basic recipe, the literature suggests that you need two or more health professionals, open communication and information sharing, understanding of roles and shared goals [22]. To a great extent, our practical experiences match the theory.

### How the West Midlands Research Collaborative (WMRC) began

A group of surgical registrars from the region met at a scientific meeting in 2007. They were united by the frustration of producing small studies at their individual units that were based on good clinical questions but were unfortunately unlikely to be published or to change practice due to the small numbers and abridged follow-up available. There was a realisation that by working together and simply carrying out each other’s projects in an identical manner at multiple units simultaneously they could create true multicentre research that blossomed into the formation of the WMRC.

After inviting all trainees in the region to get involved, a small coordinating committee was established with annual election of officers (chair, secretary, treasurer), and senior advice and guidance was obtained from Professor Dion Morton. Meetings with formal minutes were arranged and publicised via a new website and mailing list. These meetings were held on a predictable schedule (first Monday of the month) in a standard location (old hospital boardroom) in one of the central hospitals (near convenient train station), in the evening so members could attend after work. Links were established with the trials units at the University of Birmingham, who provided invaluable guidance and initial training in study design, methodology, statistics and analysis Initial projects were simple multicentre cohort studies designed to build links and cement the network, as well as to generate early some early outputs and perpetuate interest in the venture. The first RCT (the ROSSINI Trial) was designed the following year. The WMRC continues to attract new members and extend its portfolio of projects [23].

### How to manage a research collaborative so that it delivers high quality research projects

Six years experience of running the West Midlands Research Collaborative (WMRC) suggests eight fundamental principles.

1. Engage committed trainees. This brings energy, ambition, interest, determination, camaraderie, clinical knowledge (and awareness of sub-optimal practice), local knowledge (partly gained from rotation from hospital to hospital) and contacts (built up on rotation, and before it).

2. Ensure shared benefits. All those actively involved in collaborative trials and other studies are credited in final papers. Assisted by National Library of Medicine/MEDLINE/journal policies for large studies. Authorship policies are agreed upon at the start of all projects and strictly adhered to,

3. Obtain national endorsement and encouragement. Royal College support for research collaboratives in policy documents creates a favourable climate. Representation of trainee-led collaborative research groups on the National Institute for Health Research (NIHR UK) Clinical Research Networks system is developing, and already in place for Surgery.

4. Identify an inspirational mentor who is charismatic, endorses collective enterprise, lends authority and gives credibility. A mentor should bring research experience, confidence, drive, direction, discrimination. They ask the right questions, dissuade from poor ideas, delegate tasks, monitor progress, give a national presence and advocate. They should be generous about giving credit, act as honest broker in disputes and ensure fair play for participants.

5. Retain active trainee-level leadership so that committee members build experience, learn skills, share responsibility, encourage participation, support new and longstanding members. Active project management is needed for planning, implementation, monitoring and evaluation.

6. Develop local networks. Collaboratives should build strong links with local organisations such as trials units, cancer registries, patients groups. Most of these organisations welcome collaborators. Help establish other collaboratives in neighbouring regions or parallel specialities.

7. Identify supportive academics to gain partnership for funding application. These people have technical skills and knowledge, rigour, depth, experience of what is needed, and what to avoid. They can confirm/endorse that the collaborative is on the right track.

8. Deliver efficient administration (central trials office and at each local site). This takes patience, attention to detail, familiarity with governance systems, database management, stability, reliability, liaison with support staff (governance, trials unit, sponsor). Acknowledge the vital importance of experienced research nurses at local centres. They bring stability, familiarity with hospital systems/people/processes/resources, a methodical approach and tenacity (filling gaps). All projects need structure – steering groups (strategic) and operations groups (tactical) can provide this if accurate minutes are kept and disseminated.

It is not yet known if these are universal, but they are offered here as a means of stimulating debate. We have not found that the *commitment of trainee doctors* to engage in high quality research projects was an issue. The involvement of a critical mass of members from a variety of local hospital trusts ensures that implementation of projects can be expedited. One key element which ensures the involvement of trainee doctors is the clear initial guarantee that all people who contribute to a project can be certain to derive tangible *shared benefits* from it. Not only can they enhance their CVs with relevant experience, but journal publication rules permit contributors to trials to be included in article co-authorship and PubMed searchable [24]. This inclusivity enables genuine participation in studies or trials to be publicly acknowledged in a tangible way.

*National endorsement and encouragement*, such as that provided by the Royal College of Surgeons is clearly helpful [25]. Their recent report on surgical innovation recommendedthe establishment of networks and collaboration. Local leadership and the active support of an *inspirational consultant mentor* is crucial. Such a person not only promotes collective enterprise, thereby giving it credibility, but also brings research and clinical experience which saves everyone time. The mentor has extensive networks, can connect members with specialist support, helps to weed out poor ideas and gives detailed support when required. Without such a person, the risks of participation would be far greater – the presence of a senior and influential mentor ensures that members’ behaviour remains within the spirit of the collaborative. The WMRC committee membership continues to change as new trainees join and as the older members move on to established posts. However, the *active trainee-level leadership* and management of the research process is an ongoing activity. Committee members are responsible for the planning, implementation, monitoring and evaluation of all WMRC studies. Aided by the elected officers (e.g. chair, secretary, treasurer), monthly meetings are arranged, promoted and conducted in a friendly and efficient manner, and action minutes are kept so that progress can be monitored. Documents are also circulated electronically and a website is maintained.

Collaboration is not just an internal process; active engagement with existing *local networks* and resources (for example, patient participation groups, cancer registries) is beneficial to all parties. Pick up the phone. |Invite people to collaborative meetings to speak about their organisations. Take the opportunity to tell them about the collaborative’s projects and discuss areas of mutual interest. Research collaboratives should engage local *supportive academics* as much as possible, actively involving specialists in research proposals and project steering groups. In the UK, most geographical areas have university based Medical Research Council (MRC) methodology hubs (to provide specialist advice), Research Design Services (to assist with the process of identifying funding and potential research partners) and local Research and Knowledge Transfer offices or similar bodies charged with supporting nascent research projects. Similarly, registered clinical trials units will have specific expertise that collaboratives should seek in order to design the best possible research proposals and to ensure project delivery. The WMRC has strong links with each of the three clinical trials units currently in Birmingham, and works with them all, thereby benefitting from all local specialist knowledge and experience. In the UK, there are extensive frameworks of research resources which collaboratives must actively access to ensure *efficient study administration*. Previously, doctors in training may have attempted to do everything themselves. This is not only difficult, but quite unnecessary. In particular, clinical trials units have experienced trial managers, administration staff, experts in research management and governance, IT professionals who can all support funded projects. Regional National Health Service (NHS) based research networks support a large infrastructure of research nurses within local hospital trusts. These two groups of university and health service staff have invaluable experience of delivering successful studies. They have the extensive skills and experience needed to overcome obstacles, recruit patients, handle data and meet ethical requirements. Any collaborative which fails to fully engage with these resources is unlikely to succeed.

In any research project, various tasks have to be accomplished and working collaboratively facilitates this. Peer review of proposals helps to narrow research questions and enables the development of feasible research designs. All projects benefit from active monitoring in order to make progress. A stable central support system is valuable in a landscape in constant flux. Training for new and existing members enables rapid progress and saves time wasting – sharing basic information within and between projects on who, what, when, why, and how helps everyone [26].

The management literature can also provide useful guidance on running a collaborative. Some basic awareness of this is particularly valuable to medically trained researchers, who may not have been formally exposed to these ideas in their education. Teamwork is often undefined, but implies concerted effort, interdependent collaboration and shared decision making [22]. Potential consequences are job satisfaction, recognition and motivation [22]. An extensive literature on team roles has evolved. Work by Belbin and associates has identified why effective teams can be much more successful than individuals [27-29]. Nine team roles (not necessarily requiring nine or more team members – more than one role can be carried out by individuals) appear valuable. The most effective project teams have a balance of people able to carry out these roles – not too many of one type and no serious omissions. Ideally, teams will include at least one person who:

• keeps focus and engages members (coordinator),

• brings drive and momentum (shaper)

• knows people and the context (resource investigator)

• brings in-depth technical skills and knowledge (specialist)

• solves problems creatively (plant)

• plans and efficiently delivers (implementer)

• adapts and contributes (teamworker)

• works logically and impartially (monitor-evaluator)

• maintains quality (completer-finisher)

Similarly, the considerable literature on group dynamics is of interest to doctors in training who are unfamiliar with establishing and running research collaboratives [30]. Put simply, groups tend to experience various stages in their development (forming, norming, storming and performing). This is to be expected, but can nevertheless be challenging. Collaborative members need to manage these processes in order to operate effectively. From our experience, the more responsibility which collaborative members take for the whole of the research process, from conception to delivery and dissemination, the more they will learn. This sets collaboratives apart from the formal research components which are a feature of teaching in some North American medical schools. For example, the Scholarly Project at University of Pittsburgh [31] and the Research in Medicine Program at Dalhousie University [32]. The goal of collaborative members is completion of a successful project of professional interest not obtaining further qualifications. In our collaborative, advice is sought and resources are engaged, but ownership remains completely with the trainees. This adds to the pressure, but can also deliver greater satisfaction and give more confidence to continue to make successful contributions to the clinically relevant research agenda. Paradoxically, we suspect that working collectively leads to greater autonomy than supervised research.

### What has the West Midlands Research Collaborative achieved so far?

The WMRC has been up and running for six years and has progressed from modest early aspirations of undertaking multi-centre cohort studies to completing national portfolio randomised controlled trials (RCTs).

An early retrospective cohort study exploring the impact of timing of cholecystectomy after gallstone pancreatitis was been published by our group [33], and several systematic reviews have been undertaken [34-36]. The rapid emergence of parallel general surgical research groups across the UK led to the formation of the National Surgical Research Collaborative, an umbrella network whose first project was a national prospective snapshot audit of appendicectomy outcomes. This trial recruited 3326 patients from 95 centres and was published in the British Journal of Surgery [37].

The first trainee-led RCT, ROSSINI, investigating the impact of wound edge protection devices on wound infection following abdominal surgery, and completed recruitment of 769 patients from 21 centres two months ahead of schedule in January 2012 and was subsequently published in the BMJ [38]. A second multicentre RCT, DREAMS, investigated the use of dexamethasone to prevent postoperative nausea and vomiting following major gastrointestinal surgery [39]. This recruited over 1150 patients and completed nearly one-year ahead of schedule, It is currently in write-up. Several other trials are in progress from our [40], and other trainee-led research groups [41].

## Summary

Research collaboratives provide a solution to the question of how to ensure that all doctors in training gain positive experience of carrying out research with the highest scientific value, the greatest clinical relevance and the most likelihood of improving practice. Existing collaboratives have a duty to spread the message and share successes, not only their outputs but whatever they learn about the processes of planning, delivering and disseminating high quality research. In this way the various research collaboratives can work together to build on best practice and make the most of the extensive opportunities.

## Competing interests

The authors have no competing interests. All authors have completed the Unified Competing Interest form at http://www.icmje.org/coi_disclosure.pdf (available on request from the corresponding author) and declare: no support from any organisation for the submitted work [or describe if any]; no financial relationships with any organisations that might have an interest in the submitted work in the previous three years [or describe if any], no other relationships or activities that could appear to have influenced the submitted work [or describe if any].

## Authors’ contributions

GD, DB and TP drafted and redrafted the article with the help of KF and LW. GD is the guarantor for this paper. All authors read and approved the final manuscript. We are grateful to the two reviewers who improved the quality of this paper.

## Authors’ information

DB, TP, KF and LW are founder members of the West Midlands Research Collaborative and DB, KF and LW are previous chairs. TP was the lead investigator for ROSSINI. GD has worked with the WMRC and has a background in project management and management training. The paper is based on the experiences of the WMRC members and supported by citations from the relevant literature on research collaboration, planning and management. This article has been submitted on behalf of the current and previous WMRC committee members: Nick Battersby, Aneel Bhangu, Anne Gaunt, William Hawkins, Elizabeth Hepburn, Marianne Johnstone, Richard Laing, Shelagh Macleod, Paul Marriot, Tony Mak, Dion Morton, Reena Ravikumar, Caroline Richardson, Pritam Singh, Ravinder Vohra, Andrew Torrance, Haney Youssef.

## Pre-publication history

The pre-publication history for this paper can be accessed here:

http://www.biomedcentral.com/1472-6920/14/94/prepub
